# Supplementary material for: Chromothripsis during telomere crisis is independent of NHEJ, and consistent with a replicative origin
Source: Genome Res. 2019 May;29(5):737–49. doi: 10.1101/gr.240705.118 (PMC6499312; doi:10.1101/gr.240705.118)
Supplement: Supplemental Material [file supp_gr.240705.118_Supplemental_file_1.zip › contigs/annotated_contigs/DB73/contig.2.DB73_length_1048_mean_cov_6.82442748092.docx]

**DB73_length_1048_mean_cov_6.82442748092**

```
CACATCCCTTTTAAGTTGTATTCCTAGGTATTTTATTCTTTGTAGCAATTGTGAATAGGAGTTCACTCATGATTTGGCTCTCTGTCTAT
 >chr21:26809934-26810233 - E=8e-168
TATTGGTGTATAGGAATGCTTGTGATTTTTGCACGTTGATTTTGTATTCTGAGACTTTGCTGAAGTTGCTTGTTTGTTAGCTTAAGGAG

ATTTGGGGCTGAGATGATAGGGTTTTCTAAACATACAATCATGTCATCTGCAAACAGAGACTATCTGACTTCCTCTTTTCCTATTTGAA

TACCCTTTATTTCATTTTCTTGCCTGAT|TGCC|GTCTCACTGCACCTTGCCCGGGGCCGGCATCACCCCTTTTTCCTGGTGATCCACG
                             >chr21:26746106-26746859 - E=0e+00
CTGAACATGCTCCCCATCTGTGAGTCACCTGGTAGCCTTCTAGGTTACCAGATCAGCTGCGGGGATATTGCAGTGCTGGTGTTCAAGTA

ACCCTTATTTTACATCGTAATGGTCCCAAAGCCAAGAGTAGTGAGGCTGGCATGTTGTTATAATTGATTATTAGTTATTGTTGTTAAAC

TCTTATTGTGCCTAATTTATAAAATAAACATTTTTGTAGATATATACGTACAGGAAAAAGCATCACGCATACAAGGTTTGTGCTATCTG

TGGTTTCAAGCTTAGGGGTTTGGAACGTACCCCTTGAACGTAAGTAGGAACTACTGTGTTCGATTGCGTGCTCACAAGGAAAGAAAGGG

AAGAGATTGACAGAGACAGAATGAATTCTGGAAAGTCAGATTTGCCTGGGTCTGCTTAAAAGTAGGGGGAAAAAGCAGAGAGCTCTCGT

GGATACTCCTTAAGGTACTTAGTATAATGTGCCTACGATCATCTCCATTTCTGACAACCAGCAACTTCTTTCTTGTGGAGGAGTTAAGA

AGTGGTGGGTGAGGAGCAGAAGTGGGTGGGGAGAGAGGCTGGAAGTGAGAGGTCCCAAAATGCATCCTCCACTCTGGGCATAGGTAGTA

GTGTGAGGAGAGGAGGGGGAGGGGAAAGGGGAGGGGAAGGAGGGGGAGGGGAAAGGGGAGGGGAAGGAGGA
```
